# Convergent Morphological Evolution in *Silene* Sect. *Italicae* (Caryophyllaceae) in the Mediterranean Basin

**DOI:** 10.3389/fpls.2022.695958

**Published:** 2022-07-12

**Authors:** Yamama Naciri, Zeynep Toprak, Honor C. Prentice, Laetitia Hugot, Angelo Troia, Concetta Burgarella, Josep Lluis Gradaille, Daniel Jeanmonod

**Affiliations:** ^1^Unité Systématique et Médiation, Conservatoire et Jardin botaniques de Genève, Geneva, Switzerland; ^2^Plant Systematics and Biodiversity Laboratory, Department of Botany and Plant Biology, University of Geneva, Geneva, Switzerland; ^3^Department of Molecular Biology and Genetic, Faculty of Sciences, University of Dicle, Diyarbakir, Turkey; ^4^Department of Biology, Lund University, Lund, Sweden; ^5^Conservatoire botanique national de Corse, Office de l’Environment de la Corse, Corte, France; ^6^Dipartimento di Scienze e Tecnologie Biologiche, Chimiche e Farmaceutiche, Università degli Studi di Palermo, Palermo, Italy; ^7^Department of Organismal Biology, Uppsala University, Uppsala, Sweden; ^8^Jardí Botànic de Sóller, Palma, Spain

**Keywords:** species tree, species delimitation, ITS, *trn*H-*psb*A, *trn*S-*trn*G, chasmophyte, adaptation, coalescence

## Abstract

Recent divergence can obscure species boundaries among closely related taxa. *Silene* section *Italicae* (Caryophyllaceae) has been taxonomically controversial, with about 30 species described. We investigate species delimitation within this section using 500 specimens sequenced for one nuclear and two plastid markers. Despite the use of a small number of genes, the large number of sequenced samples allowed confident delimitation of 50% of the species. The delimitation of other species (e.g., *Silene nemoralis*, *S. nodulosa* and *S. andryalifolia*) was more challenging. We confirmed that seven of the ten chasmophyte species in the section are not related to each other but are, instead, genetically closer to geographically nearby species belonging to *Italicae* yet growing in open habitats. Adaptation to chasmophytic habitats therefore appears to have occurred independently, as a result of convergent evolution within the group. Species from the Western Mediterranean Basin showed more conflicting species boundaries than species from the Eastern Mediterranean Basin, where there are fewer but better-delimited species. Significant positive correlations were found between an estimation of the effective population size of the taxa and their extent of occurrence (EOO) or area of occupancy (AOO), and negative but non-significant correlations between the former and the posterior probability (PP) of the corresponding clades. These correlations might suggest a lower impact of incomplete lineage sorting in species with low effective population sizes and small distributional ranges compared with that in species inhabiting large areas. Finally, we confirmed that *S. italica* and *S. nemoralis* are distinct species, that *S. nemoralis* might furthermore include two different species and that *S. velutina* from Corsica and *S. hicesiae* from the Lipari Islands are sister species.

## Introduction

*Silene* L. is the largest genus in the family Caryophyllaceae (Greuter, 1995; Melzheimer, 1988; Bittrich, 1993) with about 870 species described around the world (Jafari et al., 2020). The genus is native to all continents apart from Australia and Antarctica, and has two main hotspots of diversity. The first, hosting more than half of the known species, is located in the Mediterranean area and the second is found in South-West Asia (Zohary, 1973; Polunin, 1980; Greuter, 1995). There are also two main centers of endemism in the Mediterranean area: Turkey and Greece, with 45 and 38% of endemic species, respectively (Coode and Cullen, 1967; Davis, 1971; Greuter, 1997; Trigas et al., 2007; Yıldız et al., 2009; Yıldız and Çırpıcı, 2013).

According to the latest revisions, the genus *Silene* is divided into three subgenera; *S.* subg. *Silene*, *S.* subg. *Behenantha* (Otth) Endl. and *S.* subg. *Lychnis* (L.) Greuter (Greuter, 1995; Jafari et al., 2020). The delimitation of the first two subgenera is supported by several earlier phylogenetic studies (e.g., Oxelman et al., 2001; Petri and Oxelman, 2011; Aydin et al., 2014a). Uncertainties, however, exist at lower taxonomic levels and the many co-existing sectional, subsectional, group and series rankings illustrate the taxonomic complexity of the genus. *Silene* section *Siphonomorpha* Otth is an example of this complexity within subg. *Silene*. The section has had a range of different taxonomic circumscriptions, and Naciri et al. (2017) showed that section *Siphonomorpha*, as defined by Coode and Cullen (1967) and Yıldız and Çırpıcı (2013), is not supported phylogenetically. Naciri et al. (2017) therefore suggested extending *Siphonomorpha* to include members of 13 previously defined sections (*Saxifragoideae*, *Coronatae*, *Tataricae*, *Chloranthae*, *Barbeyanae*, *Nanosilene*, *Otites*, *Koreanae*, *Brachypodae*, *Graminiformes*, *Longitubulosae*, *Dianthoidea*, *Holopetalae*). Within the “extended *Siphonomorpha”* group of Naciri et al. (2017), three sections were found to be monophyletic, with high support: *Paradoxae* Greuter with four species (possibly five with *S. schwarzenbergerii*), *Italicae* (Rohrb.) Schischk., with 31 species (including, for example, *S. italica* (L.) Pers. and *S. nemoralis* Waldst. and Kit.), and *Siphonomorpha* sensu Greuter (1995) with nine species, including *S. nutans* (Table 1). A fourth clade, *Giganteae*, comprising a unique species [*S. gigantea* (L) L.], still awaits a formal sectional description (Du Pasquier, 2016; Du Pasquier et al., 2017). *Italicae*, *Paradoxae*, *Siphonomorpha* and *Giganteae* are genetically close to each other but their exact phylogenetic relationships are still not well-resolved (Naciri et al., 2017; Jafari et al., 2020). Although the analysis by Jafari et al. (2020) covers a high number of *Silene* species and specimens, and further extended the definition of the *Siphonomorpha* section, the study only included around half of the species from *Italicae*, *Paradoxae*, *Siphonomorpha* and *Giganteae*. In the present study, we use the classification of Naciri et al. (2017), because the focus of the study is put on interspecific relationships within the section *Italicae*, rather than on the relationships between *Italicae* and other sections. *Italicae*, *Giganteae*, *Siphonomorpha* s.s and *Paradoxae* therefore refer to the sections defined in Naciri et al. (2017). Our study includes 73% of all species from *Italicae*, *Paradoxae*, *Siphonomorpha* and *Giganteae* (84, 80, 22, and 100%, respectively).

**TABLE 1:** List of the sampled species, their chasmophytic status, their distribution area and the number of specimens sequenced for each.

Clade	Species	Chasmophyte	Nb	Countries (Regions)
*Italicae*	*S. italica* (L.) Pers.	No	78	France, Italy, Greece, Turkey, Syria, Lebanon, Iran
	*S. nemoralis* Waldst. and Kit.	No	54	Germany, Austria, Romania, Check Republic, Slovakia, Spain, France, Italy, Serbia
	*S. badaroi* Brestr.	**Yes**	3	Southern France, Italy
	*S. mellifera* Boiss. and Reut.	No	11	Spain
	*S. coutinhoi* Rothm. and P. Silva	No	3	Spain
	*S. gazulensis* A. Galán and al.	**Yes**	3	Spain
	*S. fernandezii* Jeanm.	No	4	Southern Spain
	*S. hifacensis* Rouy	**Yes**	35	Spain (Balearic Islands, Valencia)
	*S. mollissima* (L.) Pers.	**Yes**	13	Spain (Balearic Islands)
	*S. tomentosa* Otth	**Yes**	6	Gibraltar
	*S. andryalifolia* Pomel	**Yes**	31	Northern Algeria, Northern Morocco, South Spain
	*S. patula* Desf.	No	53	Northern Algeria, Morocco, Tunisia
	*S. rosulata* Soy.-Will. and Godr.	No (on sands)	1	Algeria, Morocco
	*S. auriculifolia* Pomel	**Yes**	1	Algeria
	*S. longicilia* (Brot.) Otth	No	3	Portugal
	*S. rothmaleri* P. Silva	No	-	Portugal
	*S. hicesiae* Brullo and Signor.	**Yes**	17	Italy (Lipari Islands)
	*S. oenotriaee* Brullo	**Yes**	2	Southern Italy
	*S. nodulosa* Viv.	No	8	France (Corsica), Italy (Sardinia)
	*S. velutina* Loisel.	**Yes**	28	France (Corsica), Italy (Sardinia)
	*S. spinescens* Sibth. and Sm.	No	13	Greece (Peloponnesus, Pyrrhus)
	*S. sieberi* Fenzl	No	15	Greece (Crete)
	*S. goulimyi* Turrill	No	9	Greece (South Central Peloponnesus)
	*S. damboldtinana* Greuter and Melzh.	No	11	Northern Greece, South-eastern Albania, Southern Macedonia
	*S. cythnia* (Halácsy) Walters	No	9	Greece (Aegean islands)
	*S. galateae* Boiss.	No	5	Cyprus
	*S. niederi* Boiss.	No	5	North-western Greece
	*S. viscariopsis* Bornm.	No	-	Macedonia
	*S. waldsteinii* Griseb.	No	-	Greece, Bulgaria
	*S. splendens* Boiss.	No	-	Southwestern Turkey
	*S. astartes* Blanche ex Boiss.	No	-	Lebanon
*Giganteae*	*S. gigantea* (L.) L.	-	43	Greece, Crete, Turkey, Bulgaria
*Paradoxae*	*S. paradoxa* L.	-	12	France, Italy, Greece, Croatia, Bosnia, Macedonia
	*S. aristidis* Pomel	-	9	Algeria
	*S. fruticosa* L.	-	5	Sicily, Peloponnesus, Cyprus
	*S. sessionis* Batt.	-	-	Algeria
	*S. confertiflora* Chowdhuri	-	2	Turkey, Syria
*Siphonomorpha*	*S. nutans* L.	-	4	Spain, France, Germany, Italy, Switzerland
	*S. viridiflora* L.	-	4	Corsica, Romania, Bulgaria, Greece
	*S. berthelotiana* Webb ex H. Christ	-	-	Canary Islands
	*S. bourgaei* Webb ex H. Christ.	-	-	Canary Islands
	*S. lagunensis* Chr. Sm. ex Link	-	-	Canary Islands
	*S. nocteolens* Webb and Berthel.	-	-	Canary Islands
	*S. ponogocalyx* (Svent.) Bramwell	-	-	Canary Islands
	*S. tamaranae* Bramwell	-	-	Canary Islands
	*S. sabinosae* Pit.	-	-	Canary Islands
Total	33 species		500	

*Nb, number of sequenced samples; species that belong to one of the studied groups but that could not be included in the analysis due to a lack of material are indicated with “-.”*

The species within *Italicae* are distributed around the Mediterranean area. All the species studied so far are diploids, with a chromosome number of 2*n* = 24 (Jeanmonod, 1984a,b, 1985a; Chater et al., 1993; Yıldız and Çırpıcı, 2013) and they are hermaphrodite to gynodioecious. The western species of the section are distributed from Morocco to Tunisia in North Africa and from Portugal to Italy in Europe (Jeanmonod, 1984a,b, 1985a,1985b). The eastern species grow from the Balkan Peninsula, Greece, Western Turkey and Cyprus to Syria and, possibly, Lebanon (Du Pasquier et al., 2017). Despite several recent studies (Yıldız and Çırpıcı, 2013; Du Pasquier et al., 2017; Naciri et al., 2017) the speciation and diversification patterns of the western species of *Italicae* are still under discussion. Moreover, the evolution of the chasmophyte species within the group is poorly understood. Jeanmonod (1984a; 1984b) suggested that the seven chasmophytic species of the Western Mediterranean Basin [*S. mollissima* (L.) Pers., *S. velutina* Loisel., *S. tomentosa* Otth, *S. hicesiae* Brullo and Signor., *S. hifacensis* Rouy, *S. andryalifolia* Pomel and *S. auriculifolia* Pomel] were all derived from a chasmophytic ancestor that diverged from the ancestor of the remaining *Italicae* species during, or shortly after, the Messinian Crisis, around 5 million years ago (MYA; Gautier et al., 1994). These taxa are all characterized, among other traits, by woody and long-lived roots, numerous sterile basal rosettes (that are present at anthesis), and condensed inflorescences in a verticillaster with many flowers and short internodes. Jeanmonod (1984a; 1984b) referred to these seven western chasmophytes as the *Mollissima* group. Three additional chasmophytic species were subsequently added to this group: *S. badaroi* Brestr. (= *S. tyrrhenia* Jeanm. and Boquet; Jeanmonod and Bocquet, 1983) in South France and Italy, *S. oenotriae* Brullo in South Italy (Brullo, 1998) and *S. gazulensis* A. Galán et al. in Spain (Galán de Mera et al., 1999). Du Pasquier et al. (2017) reported that *S. gigantea*, the sole member of *Giganteae*, which is a sister group to *Italicae*, started to diversify as recently as 1.2 MYA. The results of Naciri et al. (2017) revealed that the diversification of *Italicae* was somewhat older than that of *Giganteae*—but less than 5 MYA which contradicts Jeanmonod (1984a,b) hypothesis about the divergence time of the *Mollissima* group.

Another explanation for the morphological similarity of the *Mollissima* species is that selection within harsh, rocky habitats has led to convergent evolution, with selection for similar morphologies within different lineages. Du Pasquier et al. (2017) showed that the chasmophytic characters associated with *S. gigantea* ssp. *gigantea* (i.e., flowers condensed in verticillasters and rosette leaves remaining green throughout the flowering period), appeared several times in the course of evolution. Đurović et al. (2017) showed within the *Silene saxifraga* alliance that similar morphologies were found in different, geographically shaped lineages that did not coincide with taxonomic identities. Among other processes, they highlighted the role of convergent morphological evolution to explain this pattern. Consequently, the similarities among the species of the *Mollissima* group may be explained either by a common ancestry or by convergent evolution, and resolution of the phylogenetic relationships among all the species of the section *Italicae* is required to be able to understand the evolution of the chasmophytic taxa.

An additional interesting feature of the species of *Italicae* is related to their population sizes and distributional ranges. Naciri et al. (2017) showed, on the basis of a relatively small sample size, that the species-boundaries within the western group of taxa appear to be less distinct than within the eastern group of species. The western species have, on average, wider distributional ranges than the eastern ones and it may be assumed that this correlates with higher average population effective sizes. The larger population sizes within species the more incomplete lineage sorting may be expected—blurring species boundaries (Naciri and Linder, 2015) and providing a possible explanation for the apparently fuzzier delimitation of the western species.

The present study is based on 500 specimens from *Italicae*, *Paradoxae*, *Siphonomorpha* and *Giganteae*, as defined in Naciri et al. (2017), sequenced for two plastid intergenic spacers and one nuclear ribosomal marker. The sequence data were analyzed in a Bayesian framework using the Multi-Species Coalescent (MSC) approach and we characterized each species’ distributional range using known occurrences from herbarium specimens. The study had three central aims: (1) Which relationships do the species of the *Mollissima* group have within *Italicae*—do they cluster into a single group, or do they represent unrelated species whose morphological resemblance is due to convergent evolution in rocky habitats? (2) Are species boundaries indeed less distinct in the Western Mediterranean Basin (WMB) than in the Eastern Basin (EMB) using a large sample of species and individuals? (3) Does incomplete lineage sorting (ILS) explain the observed pattern of phylogenetic variation?

## Materials and Methods

### Plant Material and Molecular Data

A total of 500 samples providing a general coverage of the distributional areas of the studied groups was used. The main sampling effort focused on *Italicae* species with 421 specimens over 500 (84.2%). The remaining specimens belong to *Giganteae* (43; 8.6%), *Paradoxae* (28; 5.6%) and *Siphonomorpha* s.s. (8; 1.6%) as outgroups. The DNA samples included in the study were extracted from the Geneva herbarium (G) specimens, except for *S. hicesiae, S. hifacensis*, *S. velutina* and *S. mollissima* for which leaf samples preserved in silica gel were used. Table 1 provides a list of the species included in the study and the number of individuals that were sequenced for each of them. For widespread species, individuals were selected from different populations so as to represent the taxon’s geographic range. When available, existing information obtained in the laboratory of the Conservatoire et Jardin botaniques de Genève (CJBG) on intraspecific genetic variation was used. We selected individuals that represent the range of variation within the species of *Silene patula* (Naciri et al., 2010) and *S. paradoxa*, *S. aristidis* and *S. fruticosa* (Leuzinger et al., 2015). The results of Prentice et al. (2003) were used to select the individuals that would be subsequently sequenced in *S. hifacensis*. Additional information about voucher specimens preserved in G, is presented in Supplementary Table 1 together with Genbank numbers.

DNA sequencing was carried out as described in Naciri et al. (2010) and Naciri et al. (2017). The plastid DNA intergenic regions *trn*H*-psb*A and *trn*S*-trn*G, and the nuclear ribosomal Internal Transcribed Spacer (ITS) were sequenced for all individuals in the CJBG laboratory and the resulting sequence data set included no missing data (Supplementary Table 1). The locus *trn*H-*psb*A was selected as it is one of the most diverse plastid ones in Angiosperms and was accordingly suggested as a suitable barcode for plants in addition to *mat*K and *rbc*L that are less diverse within species (Hollingsworth et al., 2011). The plastid *trn*S*-trn*G was also shown to be informative and polymorphic in many species of *Silene* and was used extensively in our laboratory (Naciri et al., 2010; Leuzinger et al., 2015; Du Pasquier et al., 2017 among others). The sequences produced and used for the first time (522) represented 34.8% of the total included in this study (*trn*H-*psb*A: 43.8%; *trn*S-*trn*G: 46.0%; ITS: 14.6%).

### Sequence Alignments

Multiple alignments were performed using MUSCLE implemented in Geneious version 6.1.8. (available from https://www.geneious.com) with the default options. For the three loci, we first performed the alignment on a small subset of sequences (100), then the remaining sequences were added to the existing alignment using MAFFT version 7 (Katoh et al., 2002; Katoh and Standley, 2013). Each set of the alignment was visually checked to make sure that nucleotides were consistently aligned, and alignments were manually adjusted if necessary. The two plastid loci *trn*S*-trn*G and *trn*H*-psb*A were concatenated in Geneious. Indels were coded using the approach of Simmons and Ochoterena (2000), applying the principle of “simple gap coding,” that consists in accounting for indels by replacing them in the alignment and imposing transversions instead (see Naciri et al., 2017). Substitution models were chosen on the basis of the AICc model selection criterion (Akaike’s information criterion) by running the software jModelTest2 0.1 0.10 v20160303 (Darriba et al., 2012) for both ITS and the concatenated plastid dataset. For the latter dataset jModeltest was used before and after indel coding to check for its impact on substitution model.

### Phylogenetic Analyses Under the Multi-Species Coalescent

We used the program STACEY implemented in BEAST2 (Jones et al., 2015; Jones, 2017) to estimate the species tree under the multispecies coalescent model, with no *a priori* assignment of sequences to the putative species (Drummond and Rambaut, 2007; Drummond et al., 2012; Bouckaert et al., 2014^1^).

Two input files were prepared using the STACEY template (Jones et al., 2015; Jones, 2017) implemented in BEAUTi2 (Bouckaert et al., 2014) including all the genes but with different priors. In order to minimize possible convergence problems, a starting tree obtained from the ITS data matrix by running the analyses for 50 million iterations in BEAST2, was manually added into both input xml files. For the first one, we set a general time reversible model with a gamma distribution prior and invariable sites (GTR + I + G) as the substitution model for the ITS region. For the concatenated plastid loci, we set a three-parameter model with a gamma distribution, and invariable sites (TPM1uf + I + G), with 4.0 as the “gamma category count” and 1.0 as the “shape.” We used an uncorrelated lognormal relaxed clock for both regions (mean 1.0; standard deviation 1.25) and a gamma distribution with default options on their standard deviations. The mean substitution rate of ITS was fixed to 1.0. For the plastid loci, the ploidy level was set to half (1.0) that of ITS (2.0). We set the species tree prior with the default settings. A log normal prior (mean 4.6, standard deviation 2.0) was selected for the species tree growth rate (bdcGrowthRate.t:Species). The collapse weight parameter that controls the number of possible clusters in the data set (ω; see Jones et al., 2015; Jones, 2017) was set to a Beta distribution (alpha and beta = 1.0) and a lognormal prior (mean = −7.0; standard deviation = 2.0) was set on the “popPriorScale” for population sizes. Substitution parameters for the plastid and nuclear markers were specified with an exponential distribution using the default options. A Beta prior with alpha = 1.0 and beta = 8.0 was set for the relative death parameter “relativeDeathRate.t:Species”. The second input file differed from the first one by the mean and standard deviation (1.0; 1.25, respectively) of the lognormal prior set on the “popPriorScale” for population sizes and gamma distributions specified with the default values for the substitution parameters.

The two input files were analyzed on several personal computers due to restrictions for long runs on the University cluster. For both input files, three independent chains were run for lengths of 2.1 billion generations each, by logging every 50,000 tracelog, and 2,000,000 trees. For each of the two input files the resulting tree files and log files were combined using LogCombiner after discarding the first 10% of the iterations as burn-in. We used the program Tracer version v1.6.0 (Rambaut and Drummond, 2007; Rambaut et al., 2013) to evaluate the convergence of each parameter in each run. The combined tree files were processed with Tree Annotator version 2.4.4 (Bouckaert et al., 2014). The species tree as well as the individual gene trees were visualized using the FigTree version 1.4.3 (Rambaut, 2009^2^).

### Species Delimitation

After the STACEY analyses, the species tree file was processed with the Species Delimitation Analyzer tool (SDA, see Jones et al., 2015; Jones, 2017) to estimate the content of the minimal SDA-clusters, using the following parameters: no burn-in as it was already taken into account when combining the three independent runs, 1.10^–4^ collapse height, and 1.0 similarity cut off. SDA generates a two dimensional similarity matrix, where posterior probabilities of sequence pairs belonging to the same minimal cluster are estimated. To visualize the similarity matrix and the associated SDA-clusters, we used the R code provided by Jones et al. (2015) and Jones (2017) modified by Simon Crameri. This code allows for automated pairwise similarity matrix sorting, labeling, and line drawing. We used an automatic threshold of PP = 0.01 to delineate adjacent SDA-clusters considered as putative species. SDA-clusters therefore generally contain all individuals that fall within the same clade and that have pairwise PP-values higher than 0.01 to belong to the same cluster. We then adjusted the number of final clusters (called Groups hereafter) to take into account the fact that SDA-clusters may represent genetic structure within species as well as interspecific differentiation [see Sukumaran and Knowles (2017)]. As recommended by Jones et al. (2015), we used a combination of clade supports in the species tree and of the pairwise similarity matrix to aid further considerations about species memberships. The program was run in R version 3.3.1 (R Development Core Team, 2014) and the code is available on GitHub repository^3^.

### Estimation of Distributional Ranges

Species or Group distributional ranges were estimated using the extent of occurrence (EOO) and area of occupancy (AOO). EOO is defined as the area covered by the minimum convex polygon encompassing all the known sites of occurrence of a given taxon. It might include large areas of obviously unsuitable habitats. The AOO of a taxon is defined as the area within its EOO for which its presence is certified by a record of occurrence according to a given grid. The measure reflects the fact that a taxon will not usually occur throughout its EOO but is, however, highly dependent on the sampling effort. EOO and AOO for each Group were estimated using the program GeoCat (Bachman et al., 2011), using a square mesh of 2 km^2^. We used curated georeferenced specimens taken from the GBIF repository^4^ as input data in GeoCat (see Supplementary Files). When the Groups contained two or more species, the areas of the species were calculated for all species together. In order to consider only terrestrial surfaces and avoid biases among continental species and those with a very fragmented insular distribution, the EOO of the concerned species was corrected to remove marine areas within the distributional range as follows: the polygon containing all the occurrences (EOO) produced in GeoCAT was superimposed on the official European administrative boundaries data from Eurostat (statistical office of the European Union^5^). The percentage of marine areas was then accurately estimated and the corrected EOO was reduced accordingly. The correction was necessary for two thirds of the groups and are given in Table 2. The correlation between the log of distributional ranges (either EOO or AOO) and posterior probabilities (PP) of the clades that best corresponded to each Group was then tested using Pearson’s product-moment as well as Spearman and Kendall rank correlation tests (Sokal and Rohlf, 1995) in R (R Development Core Team, 2014).

**TABLE 2:** Composition of the 16 final groups within *Italicae*, ranked from top to down in Figure 1 and SDA-clusters in Figure 2, with their associated ThetaS as an estimation of θ, extend of occurrence (EOO) and area of occupancy (AOO) in km^2^ as computed in GeoCAT.

*Italicae*	SDA-clusters	Species	Nb	Regions	PP*	EOO	EOOcor	AOO	ThetaS
Group 1	1, 2 and 3	*S. patula* + ***S. andryalifolia*** + *S. rosulata* + ***S. auriculifolia*** + ***S. tomentosa*** + ***S. gazulensis***	95	Western Mediterranean Basin	0.94	752′383	547′117 (27%)	600	3.512
Group 2	4	*S. coutinhoi*	3	Western Mediterranean Basin	0.50	250′593	250′593 (-)	588	1.333
Group 3	5	*S. mellifera*	10	Western Mediterranean Basin	0.99	271′209	264′561 (2%)	2′104	1.061
Group 4	6	***S. hifacensis*** + ***S. mollissima***	48	Western Mediterranean Basin	0.16	15′125	4′354 (71%)	172	0.901
Group 5	7	*S. nodulosa*^1^ + *S. nemoralis*^2^	22	Western Mediterranean Basin	0.37	531′420	267′733^£^ (50%) 259′007^$^ (51%)	216	1.646
Group 6	8	*S. nemoralis* ^3^	35	Eastern Europe	0.83	554′004	554′004 (-)	56	0.486
Group 7	9	*S. fernandezii*	5	Western Mediterranean Basin	0.94	842	842 (-)	96	0.480
Group 8	10	*S. nodulosa* ^4^	5	Western Mediterranean Basin	0.99	5′920	5′579 (6%)	228	0.960
Group 9	11	***S. hicesiae*** + ***S. velutina***	45	Western Mediterranean Basin	0.94	2′004	2′004 (-)	132	2.287
Group 10	12	*S. longicilia*	3	Western Mediterranean Basin	0.96	11′625	10′474 (10%)	260	1.333
Group 11	13	*S. italica* + *S. damboldtiana* + ***S. badaroi*** + ***S. oenotriae***	94	Western and Eastern Mediterranean Basin	0.81	4′248′212	2′838′218 (33%)	3,740	2.932
Group 12	14	*S. cythnia*	9	Eastern Mediterranean Basin	0.92	26′865	2′462 (91%)	192	0.368
Group 13	15	*S. goulimyi*	9	Eastern Mediterranean Basin	1.00	915	915 (-)	40	0.000
Group 14	16	*S. niederi*	5	Eastern Mediterranean Basin	1.00	24′592	22′020 (10%)	80	0.000
Group 15	17	*S. spinescens* + *S. sieberi*	28	Eastern Mediterranean Basin	0.98	80′938	25′860 (68%)	368	2.827
Group 16	18	*S. galateae*	5	Eastern Mediterranean Basin	1.00	2′112	2′112 (-)	36	0.000

*EOOcor were corrected for maritime areas, with the correction applied within brackets.*

**FIGURE 1:**
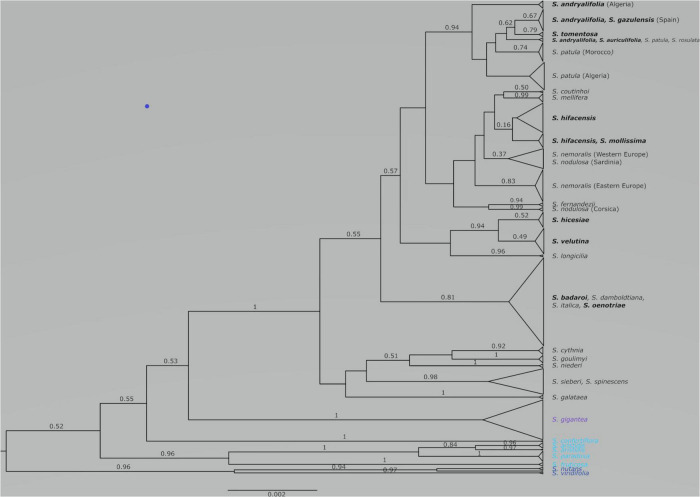
Species tree obtained from the combination of three runs using STACEY on two linked plastid regions (*trn*H-*psb*A and *trn*S-*trn*G) and one nuclear marker (ITS). With some exceptions, posterior probabilities that are equal or higher than 0.50 are given above the corresponding branches. The scale at bottom is given in substitutions/site. The chasmophytic species are indicated in bold. The species belonging to *Italicae* are shown in black whereas the species included in *Giganteae*, *Paradoxae* and *Siphonomorpha* s.s. are shown in violet, blue and dark blue, respectively.

*The PP values associated to the clades of Figure 1.

*^1^S. nodulosa from Sardinia, Italy (3 specimens); ^2^S. nemoralis from France, Italy, Spain and Bosnia (19 specimens); ^3^S. nemoralis from Austria, Romania and Slovakia (35 specimens);^ 4^S. nodulosa from Corsica, France (5 specimens).*

*^§^Corrected EOO excluding Corsica which is taken into account in Group 8 with the Corsican S. nodulosa. The chasmophyte species are given in bold.*

**FIGURE 2:**
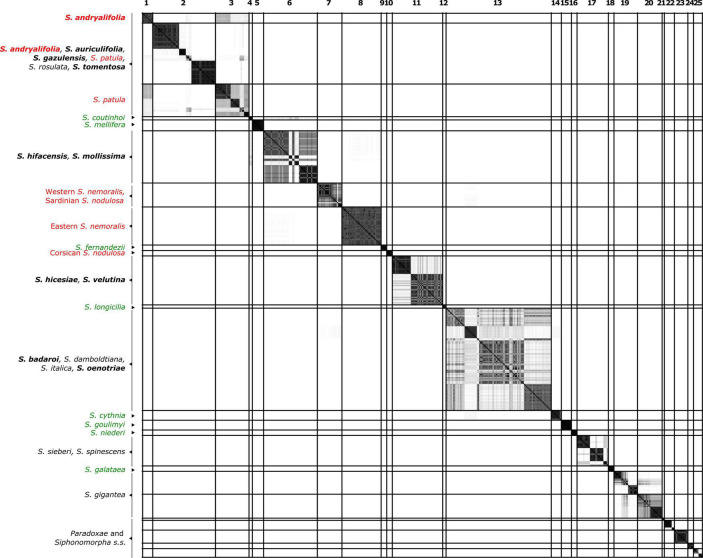
Similarity matrix showing the posterior probability that two individuals belong to the same Multi-Species Coalescent cluster (MSCC) according to the species tree of Figure 1. A black square indicates a posterior probability of 1, while no color indicates a posterior probability of 0. Specimens are given in the same order as in Figure 1. The lines delimit 26 clusters that are comprised of individuals with pairwise posterior probabilities higher than 0.01. Species found in different clusters are indicated in red, while well-delimited species are colored in green. Species in black are those found with at least one additional species in the same clade. The chasmophytic species are indicated in bold. Here, Sect. *Paradoxae* includes *S. paradoxa*, *S. confertiflora*, *S. fruticosa* and *S. aristidis* and Sect. *Syphonomorpha* s.s. includes *S. nutans* and *S. viridiflora*.

### Estimations of Effective Sizes

Species or Group effective population sizes (N_*e*_) were estimated using the metrics θ, a measure of genetic diversity, which equals 2N_*e*_μ for haploid markers, where μ is the mutation rate (Watterson, 1975). The calculation of θ assuming a constant mutation rate across taxa therefore gives access to an estimation of N_*e*_. Using the software Arlequin version 3.5 (Excoffier and Lischer, 2010), we computed θ for each Group using the chloroplast data and the Kimura-2 distance to exclude indels. We used ThetaS as an estimation of θ as it is suitable for non-recombining DNA obtained from the observed number of segregating sites among chloroplast haplotypes.

## Results

### Sequence Data and Phylogenetic Analyses

The alignment for the 500 ITS rDNA sequences was comprised of 756 nucleotide characters. For the plastid spacers, *trn*H-*psb*A and *trn*S-*trn*G, the alignments represented totals of 210 and 473 nucleotides, respectively. For each of the two input files, the three separate STACEY runs did not converge adequately for all parameters but when combined, the resulting file converged with ESS values higher than 200 for all parameters for the first input file, and higher than 300 for all parameters for the second input file (see Supplementary Table 2). Therefore, the combined runs obtained for the second input file were used in the following.

### Gene Trees

Apart from a few clades, the ITS and the pooled plastid gene trees showed congruent results at high taxonomic levels in the combined STACEY analyses. The ITS and the pooled plastid gene trees indeed revealed clades that largely correspond with *Italicae*, *Giganteae, Siphonomorpha* and *Paradoxae*. However, the plastid gene tree showed a much better agreement with the species tree topology than the ITS tree, and had much higher supports for most of the internal branches (Supplementary Figures 1, 2). In the ITS gene tree, the only species within *Italicae* that are recovered as highly supported clades are *S. goulimyi*, *S. niederi*, *S. cythnia* and *S. galataea*. In the chloroplast gene tree, *S. patula* forms a highly supported clade with the four chasmophytic species from North Africa and Spain *S. andryalifolia*, *S. auriculifolia*, *S. tomentosa* and *S. gazulensis* as well as with *S. rosulata*. Similarly *S. hifacensis* and *S. mollissima* are clustered with *S. nemoralis* from Eastern Europe with a high support (PP = 0.96).

### Species Tree for *Italicae, Giganteae, Paradoxae* and *Siphonomorpha*

The combined species tree obtained from the second input file in STACEY is illustrated in Figure 1. *Italicae* (PP = 1), *Giganteae* (PP = 1) and *Siphonomorpha* (PP = 0.96) are recovered as highly supported clades. Within *Paradoxae*, *S. aristidis* (PP = 0.84), *S. fruticosa* (PP = 1), and *S. paradoxa* (PP = 1) are clustered together with a high support (PP = 0.96) but *S. confertiflora* (PP = 1) was external to the cluster. There was poor support for the branches giving rise to *Italicae*, *Giganteae*, *Siphonomorpha* and *Paradoxae* (PP ranging between 0.52 and 0.55).

### Species Tree and Species Delimitation Within *Italicae*

Figure 2 presents the result of the species delimitation analyzer based on the species tree in Figure 1. The darker the squares, the higher the pairwise posterior probability (PP) that two individuals belong to the same SDA-cluster. The vertical and horizontal lines delineate sets of individuals that have a non-null probability of belonging to the same SDA-cluster. Within this analysis (Figure 2), 25 SDA-clusters were recovered in total, of which 18 belong to *Italicae*. Overall, twelve SDA-clusters (4, 5, 9, 12, 14, 15, 16, 18, 21, 22, 23, 24) corresponded, each, to a single species (36% of all analyzed species). Five species (15% of all species) were split into more than one group (nine SDA-clusters) whereas seven SDA-clusters (2, 6, 7, 11, 13, 17, 25) were composed of two to six species (60% of all species). For subsequent analyses we adjusted the number of clusters using the results of Figures 1, 2, keeping in mind that SDA-clusters might reflect genetic differentiation within species. Within *Italicae*, SDA-clusters 1 to 3 were merged into Group 1 (Table 2). Indeed *S*. *andryalifolia* and *S. patula* both fall into SDA-clusters 1–2 and 2–3, respectively. It is also clear from Figure 2 that SDA-clusters 1 and 2 share genetic affinities with SDA-cluster 3 with non-null pairwise posterior probabilities (PP) values. Outside *Italicae*, SDA-clusters 19 and 20 both contain *S. gigantea* specimens and should be considered as a single Group. For *Italicae*, the 18 SDA-clusters therefore correspond to 16 Groups (see Table 2).

Group 1 (PP = 0.94) contains all the North African species in addition to *S. tomentosa* from Gibraltar (PP = 0.79) and *S. andryalifolia* from both North Africa and southern Spain. Group 4 (PP = 0.16) includes *S. hifacensis* from the Balearic Islands and Alicante and *S. mollissima* which is restricted to the Balearic Islands. Group 5 (PP = 0.37) contains the Sardinian specimens of *S. nodulosa* in addition to the Western specimens of *S. nemoralis* from France, Italy and Spain. The Eastern *S. nemoralis* constitute a single group (Group 6; PP = 0.83), whereas the Corsican *S. nodulosa* are clustered in Group 8 with high support (PP = 0.99). Group 9 contains *S. hicesiae* from the Lipari Islands and *S. velutina* from Corsica (PP = 0.94) and Group 11 is comprised of *S. italica*, *S. damboldtiana*, *S. badaroi* (= *S. tyrrhenia*) and *S. oenotriae* (PP = 0.81). All the other groups represent single species (Table 2).

Within *Italicae*, the relationships between species and/or Groups remain unresolved.

### Chasmophytic Species

Within the 10 chasmophytic species of *Italicae*, those from mainland sites are typically grouped with species of open habitats within the same area. For example, *S. oenotriae*, found on inland cliffs in South Italy and *S. badaroi* (= *S. tyrrhenia*) in southern France and northwestern Italy on coastal cliffs and rocks, both cluster with *S. italica* which is widely distributed from Spain to Iran and with *S. damboldtiana* from Greece, Albania and Macedonia (Group 11, PP = 0.81; Table 2). Similarly, *S. auriculifolia* from Algeria, *S. tomentosa* from Gibraltar, *S. andryalifolia* from North Africa and Southern Spain and *S. gazulensis* from Spain cluster with *S. patula* growing inland in Morocco and Algeria (Group 1, PP = 0.94; Table 2). The former group also includes *S. rosulata* which grows on coastal sands in North Africa.

The situation is, however, different for chasmophytic species that grow on islands. *S. velutina* from Corsica and *S. hicesiae* from the Lipari Islands cluster well together (PP = 0.94; Table 2) but display no clear relationship to other species inhabiting open habitats, such as *S. nodulosa* the unique other species of *Italicae* present in Corsica. The two species *S. mollissima* (Minorca and Majorca in the Balearic Islands) and *S. hifacensis* (Ibiza in the Balearic Islands and Alicante County in Spain) are grouped in a poorly supported cluster (Group 4, PP = 0.16; Table 2) and their relationships to other mainland species are unclear (Figure 1 and Supplementary Figures 1, 2).

### Species Distributions and Effective Sizes

The correlation between the PP values and the log of the areas of occupancy and that of the corrected range extent of occurrence [log(AOO)/log(EOOCor), respectively] were computed for the 16 Groups as defined above and listed in Table 2. The maps for the 16 Groups are given in Supplementary Figure 3 and the corresponding csv files as Supplementary Files 1–5. The correction for maritime areas reduced the EOO by 2% (*S. mellifera*) to 91% (*S. cythnia;* Table 2). For AOO, the Pearson’s product-moment correlation and the two rank tests were negative and non-significant (cor = −0.111, *P* = 0.682; Kendall’s rank correlation tau = −0.233, *P* = 0.228; Spearman’s rank correlation rho = −0.350, *P* = 0.184). For EOOCor, even more negative but still non-significant correlation coefficients were obtained (cor = −0.222, *P* = 0.408; tau = −0.300, *P* = 0.116; rho = −0.406, *P* = 0.120). The PP values were also negatively correlated with θ estimates (cor = −0.064, *P* = 0.813; tau = −0.350, *P* = 0.064; rho = −0.456, *P* = 0.078). AOO and EOOCor were positively and significantly correlated with θ estimates (Table 3).

**TABLE 3:** Pairwise correlations between the area of occupancy (AOO), the extent of occurrence (EOOCor), the posterior probabilities (PP) and ThetaS as an estimation of θ for the Groups of Table 2.

		log(AOO)	Log(EOOCor)	ThetaS
PP	Pearson	−0.111^ns^	−0.222^ns^	−0.064^ns^
	Kendall’s tau	−0.233^ns^	−0.300^ns^	−0.350^ns^
	Spearman’s rho	−0.350^ns^	−0.406^ns^	−0.456^ns^
log(AOO)	Pearson	−	0.674**	0.667**
	Kendall’s tau	−	0.629*	0.583**
	Spearman’s rho	−	0.533**	0.779***
log(EOO-Cor)	Pearson			0.539*
	Kendall’s tau			0.417*
	Spearman’s rho			0.629*

^ns^ for P > 0.05; * for P > 0.05; ** for P < 0.01 and *** for P < 0.001.

## Discussion

### Well Defined Sections but Unclear Relationships Among Them

Clear morphological characterizations of *Italicae*, *Paradoxae*, *Giganteae* and *Siphonomorpha* s.s. have been given by Chowdhuri (1957), Jeanmonod and Mascherpa (1982), Greuter (1995) and Du Pasquier (2016), among others. Eleven species belonging to these four groups (*S. italica, S. nemoralis, S. tomentosa, S. andryalifolia, S. gazulensis, S. rothmaleri, S. waldsteinii*, *S. paradoxa*, *S. fruticosa, S. gigantea* and *S. nutans*) were included in one of the most complete published phylogenies of the Caryophyllaceae based on ITS and five plastid loci (Greenberg and Donoghue, 2011). However, the genetic relationships among these species, as well as the relationships among the different sections remained unresolved in that study. In the latest phylogeny of the genus *Silene*, based on ITS and the plastid loci *rps*16, Jafari et al. (2020) increased the number of species analyzed for the same groups to 22, i.e., ten for *Italicae* (*S. italica, S. nemoralis* from Austria, *S. nodulosa* from Corsica, *S. longicilia, S. andryalifolia, S. hifacensis, S. damboldtiana, S. cythnia, S. viscariopsis, S. waldsteinii*), three for *Paradoxae* (*S. paradoxa*, *S. aristidis*, *S. fruticosa*), and eight for *Siphonomorpha* s.s. in addition to *S. gigantea* with a similar conclusion—no clear relationships emerged among the groups. The present study, however, confirms the consistency of *Italicae*, *Giganteae* and *Siphonomorpha* and, to a lesser extent, *Paradoxae*, as defined by Naciri et al. (2017). *Silene confertiflora* is always recovered as a highly supported clade in our analysis (Figure 1). Nevertheless the relationships of this species with sections *Italicae* or *Giganteae* is poorly understood and its placement remains unresolved in our study.

Despite the fact that a much greater number of specimens and species were analyzed in the present study compared to Naciri et al. (2017), the relationships among the four sections remain poorly understood. The low resolution among these sections may reflect a rapid diversification that occurred within a short period of time. In our study, the unclear relationships among sections cannot be attributed to a low analysis convergence because all the parameters gave ESS values above 300 (Supplementary Table 2). The parameters that displayed the lowest ESS values and were the latest to converge are the prior (ESS = 302), the birth-death-collapse model (ESS = 310) and the number of clusters (ESS = 345). These parameters are among the ones known to converge sometimes barely with STACEY (Jones, 2017). The birth-death-collapse prior, and the number of clusters are related to populations and species dynamics, which are difficult to estimate, but should not impact the support of deep branches. Jones (2017) pointed out that the birth-death-collapse prior depends strongly on the number of clusters, which might explain that they both exhibit among the lowest ESS values. In our analyses, the number of clusters was not constrained in the priors but the recovered SDA-clusters nevertheless mostly agreed with already described species. Another explanation for the ESS of the prior remaining among the lowest ones could be due to the dataset lacking enough information, i.e., with a too low number of substitutions. Disentangling the two explanations is difficult, however, and including data from more genes, using NGS techniques for instance (Toprak et al., unpublished results) might improve the observed pattern.

### A Wide Range of Support for Species Boundaries Within *Italicae*

In STACEY, gene trees and species trees are estimated simultaneously. This is not the case for other methods that require a fixed guide tree and then estimate the fit of that particular tree with the proposed gene trees (Aydin et al., 2014b). Under STACEY, any change to the species tree, or to the different gene trees during the MCMC move, may improve or decrease the concordance between the gene trees and the species tree. The way STACEY produces the species tree, however, minimizes the influence of the prior settings on the outcomes of the analyses. In contrast to programs such as *BEAST, STACEY uses no prior knowledge about samples’ assignment to species—reducing the impact of any prior knowledge (or any assignment error) on the results. Our analyses ended up with a variety of results—from genetically well-delimited species to species that present fuzzy genetic boundaries, or species that are divided into different lineages. Generally speaking, our analyses led to a better overall resolution than other studies such as Greenberg and Donoghue (2011) for instance, who used more genes than we did (ITS, *mat*K, *ndh*F, *trn*L-*trn*F, *trn*Q-*rps*16, and *trn*S-*trn*fM). This might be due to several factors: first *trn*H-*psb*A is one of the most diverse chloroplast gene among angiosperms but was seldom used in other *Silene* studies. We furthermore coded the indels which increases the informativeness of the locus. Second, the MSC approach takes into account that genes might be incongruent among them because they have their own independent histories and signal, which might not reflect organismal phylogeny. This means that part of the incongruence among genes is considered as “normal” under the MSC and STACEY deals with that when estimating the species tree. Third, in this paper, the support is given for putative species not for individual genes. Finally, the fact that we take into account the intraspecific diversity might also explain why we obtained a better resolution.

Within the *Italicae* section, AOO and EOO as well as ThetaS were used as proxies for estimating effective population sizes (N_*e*_). Significant positive correlations were found among those three parameters suggesting that species with low effective sizes indeed inhabit smaller ranges overall. However, we could not confirm that these lower effective sizes translate into higher PP-values as all correlations with PP were negative, as expected, but still non-significant. This lack of significance despite high correlations (as the one between PP and ThetaS found to be −0.456) might have different origins: first, a low test power as only 16 pairs of observations were taken into account; second, the fact that EOO and AOO are imperfect estimates of the species range and occupancy as they depend of the species knowledge and the collection records that can be found in public repositories such as GBIF; and third, the fact that ThetaS is also an imperfect estimation of the species effective size as it assumes a constant mutation rate across Groups and was designed for populations rather than for species or group of species. It would be interesting to test for similar correlations on a higher number of species to confirm on *Silene* the theoretical prediction that small effective population sizes lead to more rapid and complete lineage sorting (Naciri and Linder, 2015).

### The Widely Distributed Species *S. italica*, *S. patula* and *S. nemoralis*

Within *Italicae*, three species are widely distributed: *S. italica* (EOO = 3,025,801 km^2^), *S. patula* (EOO = 443,117 km^2^) and *S. nemoralis* (EOO = 418,617 km^2^). *Silene nemoralis* is placed into two different groups that agree well with geography (Group 5 from France, Italy, Spain and Bosnia including *S. nodulosa* from Sardinia and Group 6 from Austria, Romania and Slovakia) that both have moderate to low supports (PP = 0.37 and 0.83, and ThetaS = 1.646 and 0.486, respectively). The relationships between Groups 5 and 6 and other taxa are not well assessed, but the split of *S. nemoralis* into two genetic entities suggests either a high level of incomplete lineage sorting within a single species or the existence of two putative species that diverged recently, one in the Western Mediterranean Basin and the other in Eastern Europe. The assignment of *S. nemoralis* to two different clusters is consistent with the fact that the species has previously been subdivided, on the basis of morphological characters, into three different taxa: two western (*S. crassicaulis* Willk. and Costa in the Pyrenees and *S. nemoralis* var *pedemontana* Burnat and Barbey in the French Maritime Alps and Northern Italy) and one (*S. nemoralis s.str.*) restricted to Eastern Europe (Jeanmonod, 1985b). Therefore we could consider that *S. nemoralis* is the species actually found in Eastern Europe whereas *S. crassicaulis* is that of the Western Mediterranean Basin.

Unlike *S. nemoralis*, *S. italica* and *S. patula* each fall within a single group that includes several species, with moderate to high clade supports (PP = 0.81 and PP = 0.94, respectively). For *S. patula*, Group 1 includes 5 other species (*S. andryalifolia*, *S. auriculifolia*, *S. tomentosa*, *S. gazulensis* and *S. rosulata*), whereas for *S. italica*, Group 11 also includes *S. damboldtiana*, *S. oenotriae* and *S. badaroi*. These two groups include chasmophytic and non-chasmophytic species and their ThetaS estimations are the two highest (3.512 and 2.932, respectively).

Du Pasquier et al. (2017) reported that *S. gigantea* started to diversify around 1.2 MYA. According to Figure 1, similar or even younger ages may be inferred for the diversification of Groups 1 and 11. Therefore, if high effective population sizes can be assumed in taxa that, in addition, have had a recent history of diversification, it can be hypothesized that the diffuse species-boundaries within those groups are unlikely to be resolved by simply analyzing a higher number of genes.

### The Chasmophytic Species of the *Mollissima* Group: A Case of Morphological Convergence

The *Mollissima* group as defined by Jeanmonod (1984b) is clearly polyphyletic and a common ancestor exclusively for all its species is not found. The switch between open rocky and chasmophytic habitats appears to have occurred several times within *Italicae*: all the chasmophytic species of Southern Spain (*S. andryalifolia*, *S. gazulensis*, *S. tomentosa*) and Northern Africa (*S. auriculifolia* and *S. andryalfolia*) share a common ancestor with *S. patula*, assessed by the high support found for the clade comprising all five species (Group 1; PP = 0.94). This is the first time that such a relationship between chasmophytic species and non-chasmophytic species has been confirmed with molecular data within *Italicae*. Our results also show for the first time that *S. velutina* and *S. hicesiae* diverged from the same ancestor, as assessed by the strong support of the clade including both species (Group 9; PP = 0.94). For that species pair, it is unclear which non-chasmophytic taxon is their closest relative. Similarly, the two species *S. mollissima* and *S. hifacensis* also share a clear genetic similarity according to Figure 2, although their group displays no support (Group 4, PP = 0.16). As for the preceding case, it is difficult to link them clearly with any other species although they show some genetic affinities with *S. coutinhoi* (Figure 2). The remaining two chasmophytic species (*S. oenotriae* and *S. badaroi*) that were not formally included in the *Mollissima* group of Jeanmonod (1984b) are clearly related to *S. italica* (Group 11, PP = 0.81). The *Mollissima* group has therefore no evolutionary significance in terms of shared ancestry but rather comprises species that seems to have independently adapted to chasmophytic conditions. Cultivation experiments are needed to assess the extent to which, at least in some cases, the morphological differentiation in chasmophytic species may partly reflect phenotypic plasticity. For example, *S. oenotriae* and *S. badaroi* have similar inflorescence characteristics to *S. italica*. When cultivated, *S. badaroi* (= *S. tyrrhenia*) displayed only attenuated characteristics of a chasmophytic plant (Jeanmonod and Bocquet, 1983), suggesting that a degree of plasticity may be involved in the morphological differentiation between the chasmophyte and the non-chasmophyte *S. italica*.

Overall, our results suggest that chasmophytic species, or at least some of them, have derived from geographically neighboring species that occur in open habitats. Chasmophytic species are usually found in restricted rocky places or cliffs and as small populations that are highly susceptible to genetic drift. In the case of *S. oenotriae* and *S. badaroi*, the process of speciation appears to be ongoing and the fact that their distributional ranges overlap with that of *S. italica* suggests that gene flow might remain between the chasmophytes and the non-chasmophyte. Both chasmophytes share the same single plastid haplotype (A1B1) and almost identical ITS sequences to that of *S. italica*. Depending on the species concept used, the two chasmophytic species could be regarded as ecotypes of *S. italica* rather than species. Our results suggest that the ecological divergence may have predated the reproductive isolation of the taxa (Coyne and Orr, 2004; Schemske, 2010). This conclusion would match that of Karrenberg et al. (2018) who demonstrated that in the speciation process between *S. latifolia* and *S. dioica*, extrinsic barriers associated with adaptive ecological divergence had more importance than intrinsic postzygotic barriers in building effective reproductive isolation.

The case of *S. patula*, and its associated chasmophyte taxa is more complex. Within Group 1, the relationships among the different clades are not resolved. From Figure 2, it is however noticeable that *S. patula* from Algeria has some genetic affinities with *S. andryalifolia* and *S. auriculifolia* from the same country, whereas *S. andryalifolia* from Spain is clustered with *S. tomentosa* and *S. gazulensis* from Spain. Naciri et al. (2010) showed that Eastern Algeria hosts most of the genetic diversity within *S. patula*, while the Moroccan populations are homogeneous. Genetic homogeneity within Morocco was attributed to a rapid and recent colonization of the country from Algeria, with associated plastid haplotype surfing (Klopfstein et al., 2006). According to this scenario, *S. andryalifolia* from North Africa would have diverged *in situ* before crossing the Straits of Gibraltar, and subsequently giving rise to other chasmophytic species in the Iberian Peninsula. The case of the restricted endemic from Oran, *S. auriculifolia*, is difficult to resolve, as only one accession could be included in the analyses. The species exhibits a single plastid haplotype (A15B16) that is also found in two North Moroccan specimens of *S. patula* with which it clusters, together with one specimen of *S. rosulata* from Alger and one specimen of *S. andryalifolia* from Tizi Ouzou.

Although the whole group of North-African and Iberian species is well supported (PP = 0.94; Group 1), unresolved relationships with the remaining species make it difficult to establish the relationships between this group and other species of *Italicae*. The unresolved relationships for the remaining chasmophytic species (*S. velutina*, *S. hicesiae*, *S. hifacensis*, *S. mollissima*) do not allow suggestions about divergence scenarios. However, the two examples detailed above (*S. italica* and *S. patula* and their allied species), suggest that the chasmophytic form corresponds to a morphological convergence driven by independent adaptation to cliffs or rocky habitats. A similar morphological convergence is also found in *Paradoxae* where *S. aristidis* and *S. sessionis* display chasmophytic characteristics while *S. paradoxa* does not (Leuzinger et al., 2015). Confirming convergent evolution among species from different areas will require the identification of the genes associated with the chasmophytic form and their comparison among species.

Plasticity and morphological convergence are common in *Silene*, perhaps explaining the difficulty encountered when trying to find infra-generic classifications (Naciri et al., 2017; Jafari et al., 2020). For instance, Du Pasquier et al. (2017) highlighted a strong morphological convergence leading to the chasmophytic form in different genetic lineages all described under *S. gigantea* subsp. *gigantea*. Similarly, Đurović et al. (2017) showed that morphological convergence partly explains why taxonomic delineation based on morphology does not match with genetics in the *Silene saxifraga* alliance. As in *S. andryalifolia* or *S. nemoralis* the genetic differentiation is rather geographically than morphologically structured in that group.

### *Silene italica* and *S. nemoralis* Are Two Different Species

*Silene nemoralis* is one of the few species within *Italicae* (with *S. damboldtiana*) that is biennial. This character discriminates it from *S. italica* with which it has been often lumped. The Flora of Italy (Pignatti, 1982) used to consider *S. nemoralis* as a subspecies of *S. italica* however, in the recent second edition of the Flora (Pignatti et al., 2017), the two taxa are now treated as separate species. Our results clearly confirm that *S. nemoralis* and *S. italica* can be regarded as different species. Their distributional ranges do not overlap, and the species occupy different habitats. *Silene nemoralis* grows in the forest understory and in cool sites whereas *S. italica* is found in open and drier habitats. Because the relationships within *Italicae* are unresolved, it is difficult to say whether the ancestor of *Italicae* was perennial or biennial. A better resolved phylogeny based on more data would be needed to conclude on the ancestral state of perennials versus biennials within *Italicae*.

### A High Level of Incomplete Lineage Sorting

This study highlights the impact of incomplete lineage sorting in recently diverging lineages (Naciri and Linder, 2020). For both gene trees (and in the resulting species tree) *Italicae*, *Giganteae*, *Paradoxae* and *Siphonomorpha* s.s. are recovered as highly supported clades but the resolution within the clades is much higher with the chloroplast genes than with ITS. This can be related to the respective effective sizes of the two types of genes—which is twice as high for ITS compared to the chloroplast (a diploid nuclear gene versus haploid chloroplast genes). Therefore, as observed here, more incomplete lineage sorting is expected for ITS than for the chloroplast genes (Naciri and Linder, 2015). Another explanation for conflicting results between ITS and the plastid markers might be the possible incomplete ITS concerted evolution mode after recent hybridization events (Nieto Feliner and Rosselló, 2007) leading to unresolved topologies. In our case, ITS gave almost no resolution at the species level, except for three species of the Eastern Mediterranean basin that have PP values of 1, restricted distribution ranges and no within-species diversity as captured by ThetaS. This is in contradiction with previous finding in *Silene* (Rautenberg et al., 2010) and with the general expectation that nuclear genes will provide better assignment to species than chloroplast genes (Naciri et al., 2012)—one of the reasons why ITS has been considered for species assignment in addition to the chloroplast barcoding genes (China Plant BOL Group et al., 2011; Hollingsworth et al., 2011). In the case of *Italicae*, it is anticipated that a completely resolved phylogeny might be difficult to obtain, even with a large number of genes, as a result of the confounding effects of large effective population sizes for some species and a recent and fast diversification history, both resulting in a high level of incomplete lineage sorting that blurs species delimitation and relationships (Naciri and Linder, 2015). The relationships among species might be difficult to recover because of the recent radiation of *Italicae* in the Mediterranean region. This hypothesis will, however, have to be tested using a higher number of genes using NGS technologies.

## Data Availability Statement

The datasets presented in this study can be found in online repositories. The names of the repository/repositories and accession number(s) can be found in the article/Supplementary Material.

## Author Contributions

DJ and YN designed the project. YN produced the data. YN and ZT analyzed the data and wrote the manuscript. HP, DJ, CB, and AT revised the different versions of the manuscript. HP, LH, CB, AT, and JG provided samples for the study. All authors contributed to the article and approved the submitted version.

## Conflict of Interest

The authors declare that the research was conducted in the absence of any commercial or financial relationships that could be construed as a potential conflict of interest.

## Publisher’s Note

All claims expressed in this article are solely those of the authors and do not necessarily represent those of their affiliated organizations, or those of the publisher, the editors and the reviewers. Any product that may be evaluated in this article, or claim that may be made by its manufacturer, is not guaranteed or endorsed by the publisher.

## Footnotes

1
https://www.beast2.org/

2
http://beast.bio.ed.ac.uk

3
https://github.com/scrameri/smtools/tree/master/SpeciesDelimitation

4
https://www.gbif.org/

5
https://ec.europa.eu/eurostat/web/gisco/geodata/reference-data/administrative-units-statistical-units/countries

## Supplementary Material

Supplementary Figure 1 Gene tree obtained from the combination of three runs using STACEY on two linked plastid regions (*trn*H-*psb*A and *trn*S-*trn*G). Posterior probabilities that are equal or higher than 0.50 are given above the corresponding branches. The scale at bottom is given in substitutions/site. The chasmophytic species are indicated in bold. For some species, the geographic origin is indicated between brackets. The species belonging to *Italicae* are shown in black whereas the species included in *Giganteae*, *Paradoxae* and *Siphonomorpha* s.s. are shown in violet, blue and dark blue, respectively.

Supplementary Figure 2 Gene tree obtained from the combination of three runs using STACEY on and one nuclear marker (ITS). Posterior probabilities that are equal or higher than 0.50 are given above the corresponding branches. The scale at bottom is given in substitutions/site. The chasmophytic species are indicated by bold. The species belonging to *Italicae* are shown in black whereas the species included in *Giganteae*, *Paradoxae* and *Siphonomorpha* s.s. are shown in violet, blue and dark blue, respectively.

Supplementary Figure 3 Distribution maps with the estimations of extents of occurrence (EOO) delimited by a blue line and the occurrences given as red dots according to GeoCAT for the 16 Groups of Table 2 and the files given as Supplementary data.

Supplementary Table 1 List of the specimens used in this study together with their collection and lab numbers, the collector name, country of origin and Genbank number accessions for the plastid *trn*H-*psb*A, *trn*S-*trn*G and the nuclear ribosomal Internal Transcribed Spacer (ITS).

Supplementary Table 2 ESS values for the different parameters in the combined STACEY analysis.

Supplementary File 1 Coordinates obtained from GBIF for Groups 1, 2, and 3.

Supplementary File 2 Coordinates obtained from GBIF for Groups 4, 5, 6, and 7.

Supplementary File 3 Coordinates obtained from GBIF for Groups 8, 9, and 10.

Supplementary File 4 Coordinates obtained from GBIF for Group 11.

Supplementary File 5 Coordinates obtained from GBIF for Groups 12, 13, 14, 15, and 16.
