# Erratum: Convergent morphological evolution in *Silene* Sect. *Italicae* (Caryophyllaceae) in the Mediterranean Basin

**DOI:** 10.3389/fpls.2023.1182244

**Published:** 2023-03-20

**Authors:** Frontiers Production Office

**Affiliations:** Frontiers Media SA, Lausanne, Switzerland

**Keywords:** species tree, species delimitation, ITS, *trn*H-*psb*A, *trn*S-*trn*G, chasmophyte, adaptation, coalescence

An omission to the funding section of the original article was made in error. The following sentence has been added: “Open access funding was provided by the University of Geneva”.

The original version of this article has been updated.

