# Supplementary figures and images for: Convergent Morphological Evolution in Silene Sect. Italicae (Caryophyllaceae) in the Mediterranean Basin
Source: Front Plant Sci. 2022 Jul 12;13:695958. doi: 10.3389/fpls.2022.695958 (PMC9319200; doi:10.3389/fpls.2022.695958)

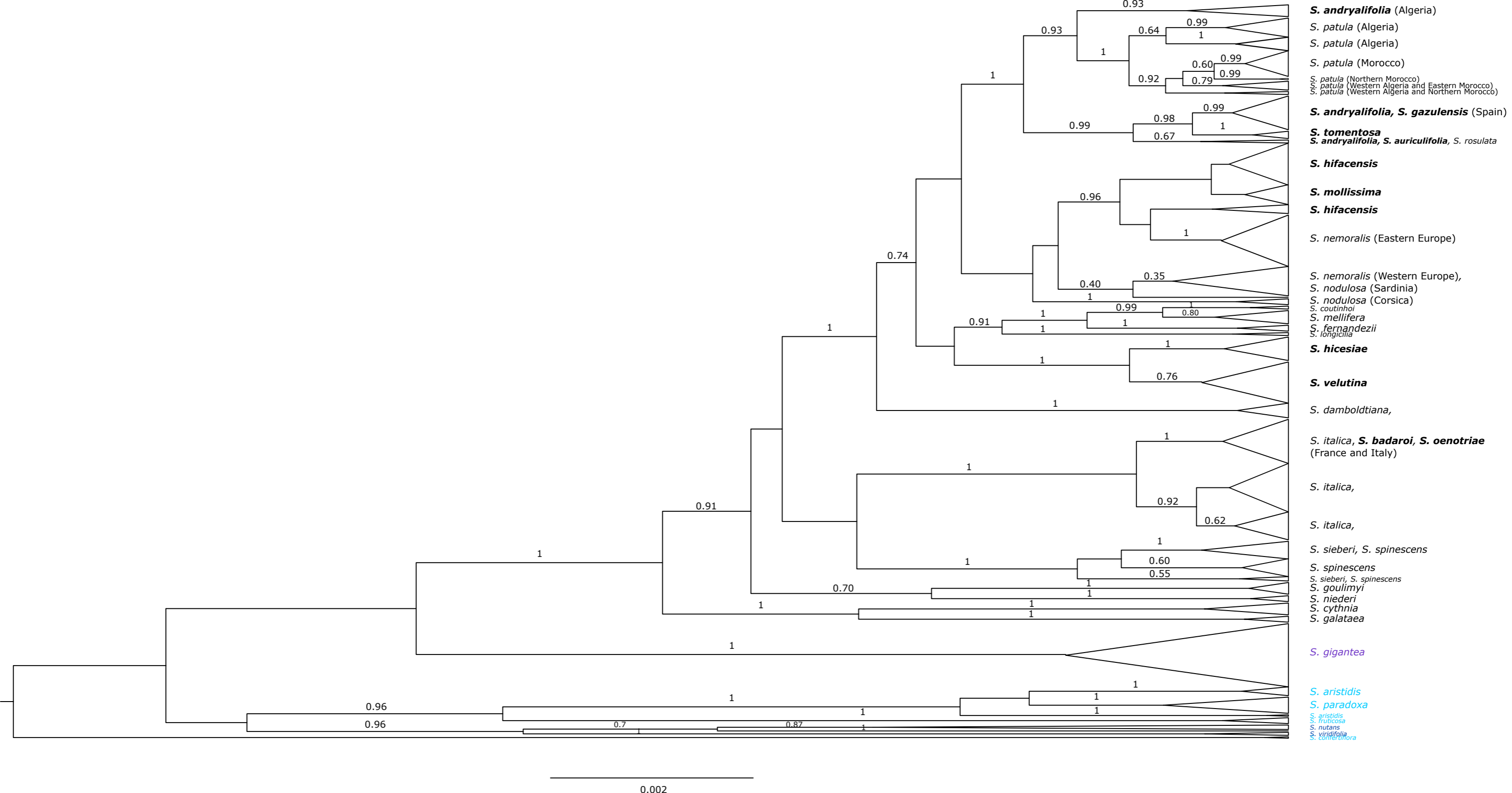

Supplement: Supplementary Figure 1 — Gene tree obtained from the combination of three runs using STACEY on two linked plastid regions (trnH-psbA and trnS-trnG). Posterior probabilities that are equal or higher than 0.50 are given above the corresponding branches. The scale at bottom is given in substitutions/site. The chasmophytic species are indicated in bold. For some species, the geographic origin is indicated between brackets. The species belonging to Italicae are shown in black whereas the species included in Giganteae, Paradoxae and Siphonomorpha s.s. are shown in violet, blue and dark blue, respectively. [file Image_1.pdf]

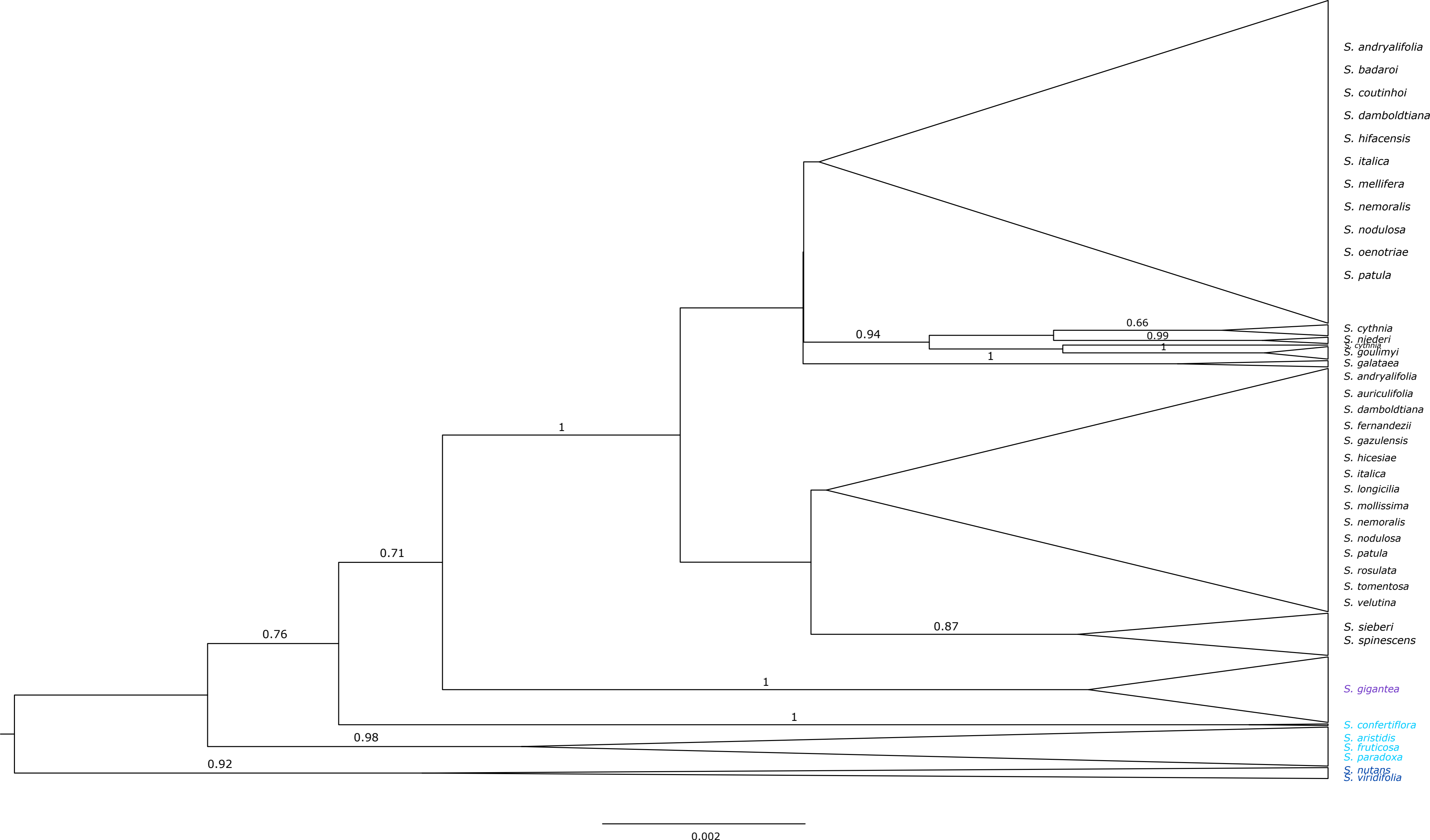

Supplement: Supplementary Figure 2 — Gene tree obtained from the combination of three runs using STACEY on and one nuclear marker (ITS). Posterior probabilities that are equal or higher than 0.50 are given above the corresponding branches. The scale at bottom is given in substitutions/site. The chasmophytic species are indicated by bold. The species belonging to Italicae are shown in black whereas the species included in Giganteae, Paradoxae and Siphonomorpha s.s. are shown in violet, blue and dark blue, respectively. [file Image_2.pdf]
