# Supplementary material for: Convergent Morphological Evolution in Silene Sect. Italicae (Caryophyllaceae) in the Mediterranean Basin
Source: Front Plant Sci. 2022 Jul 12;13:695958. doi: 10.3389/fpls.2022.695958 (PMC9319200; doi:10.3389/fpls.2022.695958)
Supplement: Supplementary Figure 3 — Distribution maps with the estimations of extents of occurrence (EOO) delimited by a blue line and the occurrences given as red dots according to GeoCAT for the 16 Groups of Table 2 and the files given as Supplementary data. [file Image_3.pdf]

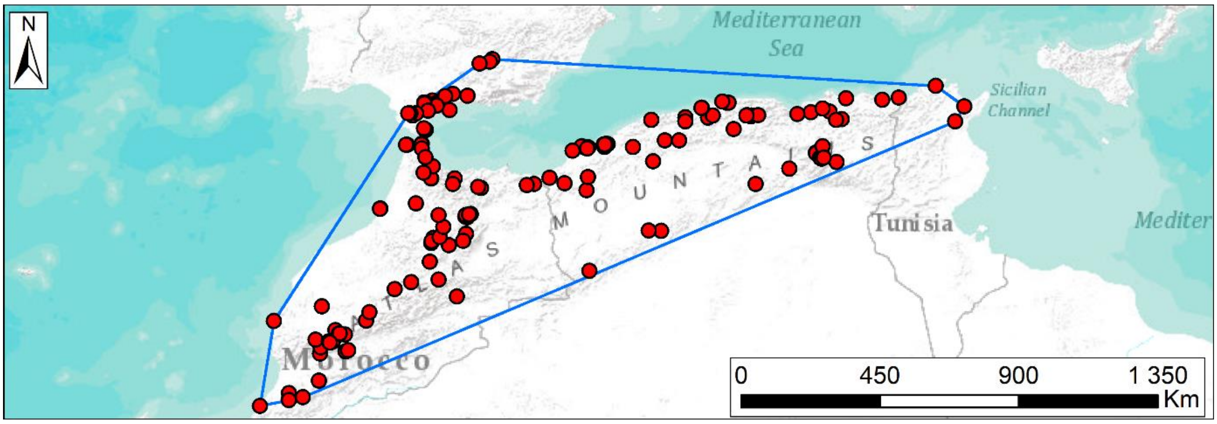

Group 1. *Silene patula*, *S. andryalifolia*, *S. auriculifolia*, *S. rosulata*, *S. tomentosa* and *S. gazulensis*

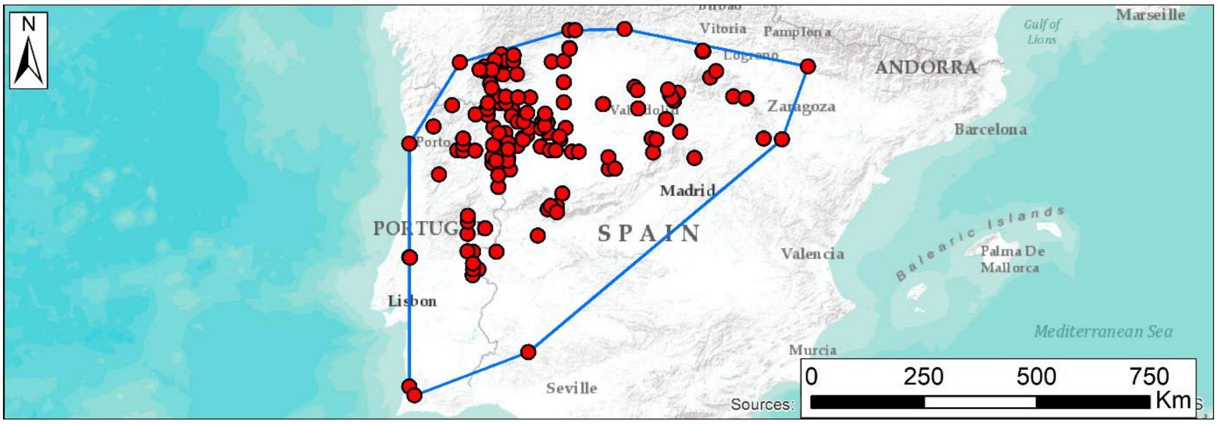

Group 2. *Silene coutihoi*

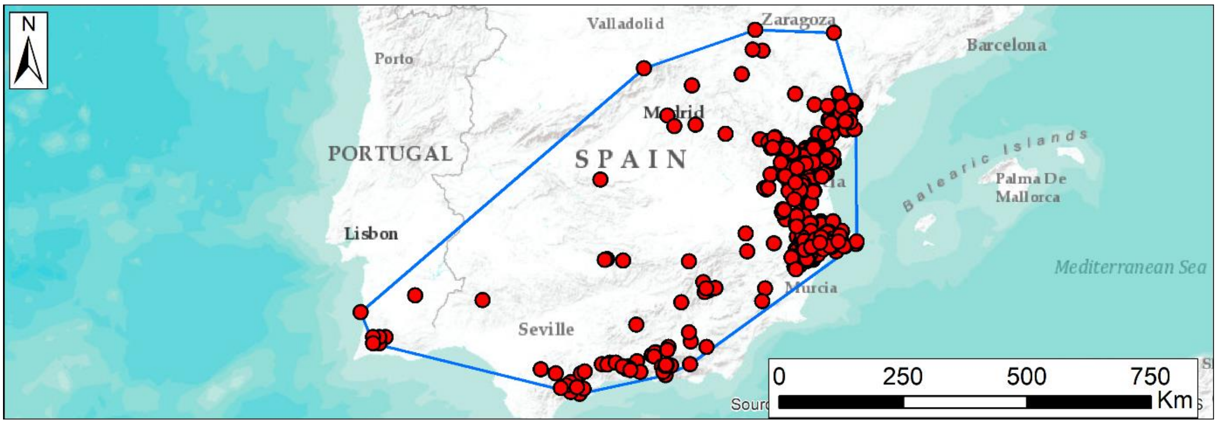

Group 3. *Silene mellifera*

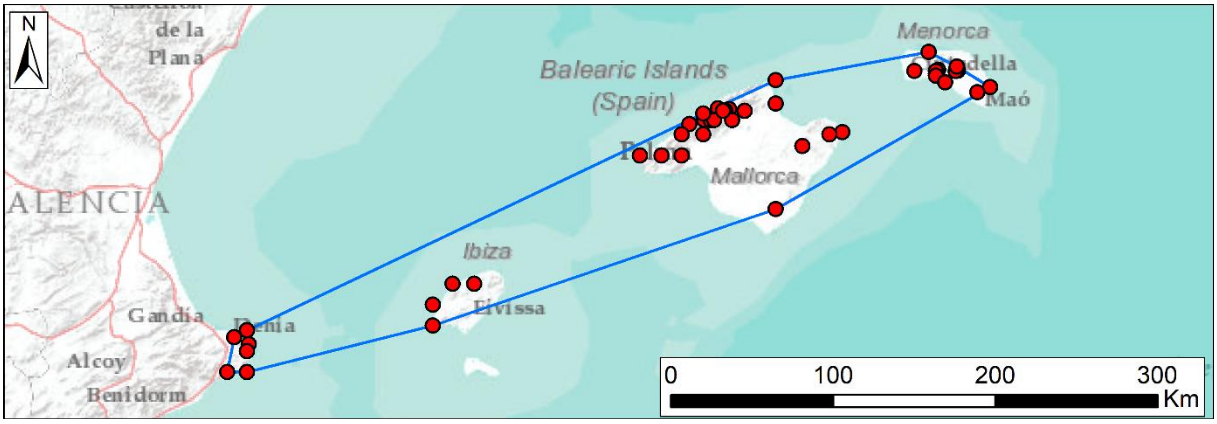

Group 4. *Silene mollissima* and *S. hifacensis*

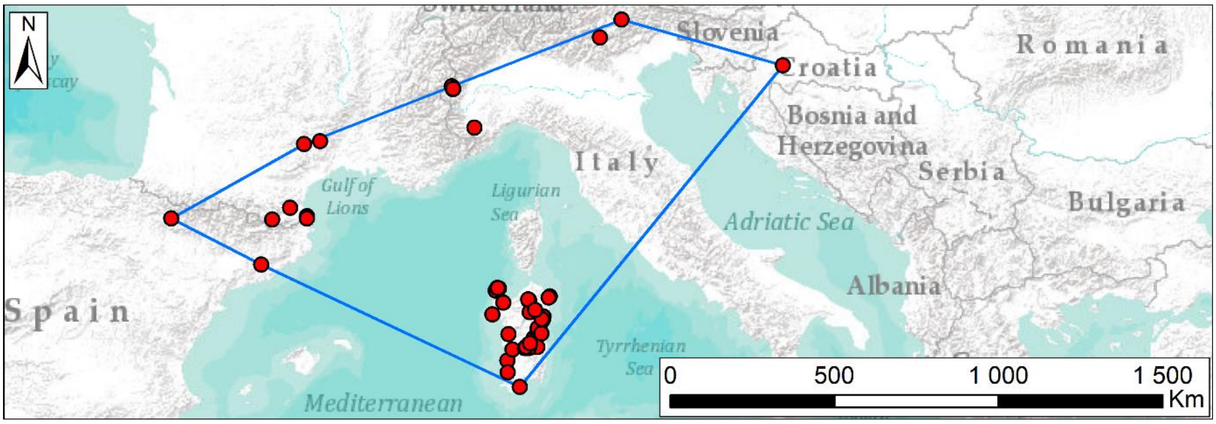

Group 5. *Silene nemoralis* (West) and *S. nodulosa* (Sardinia)

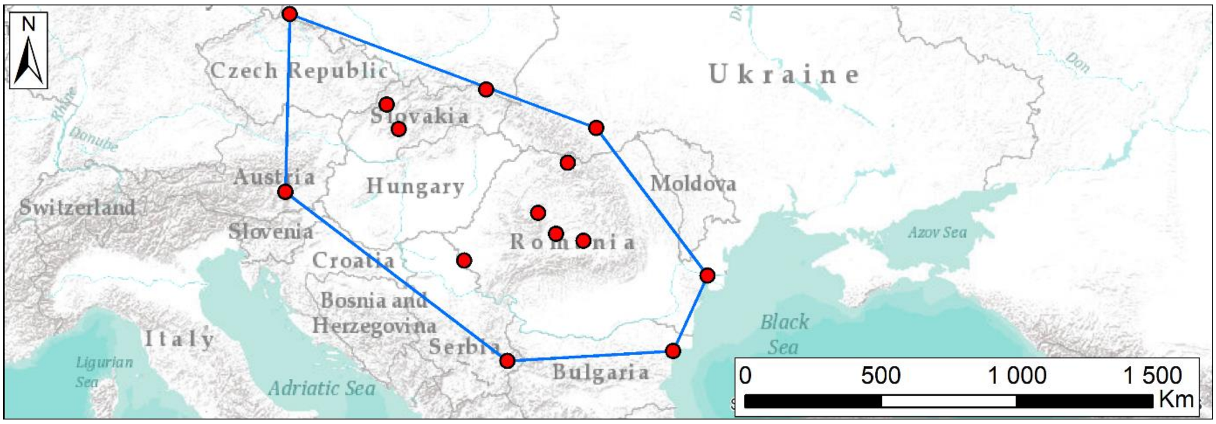

Group 6. *Silene nemoralis* (East)

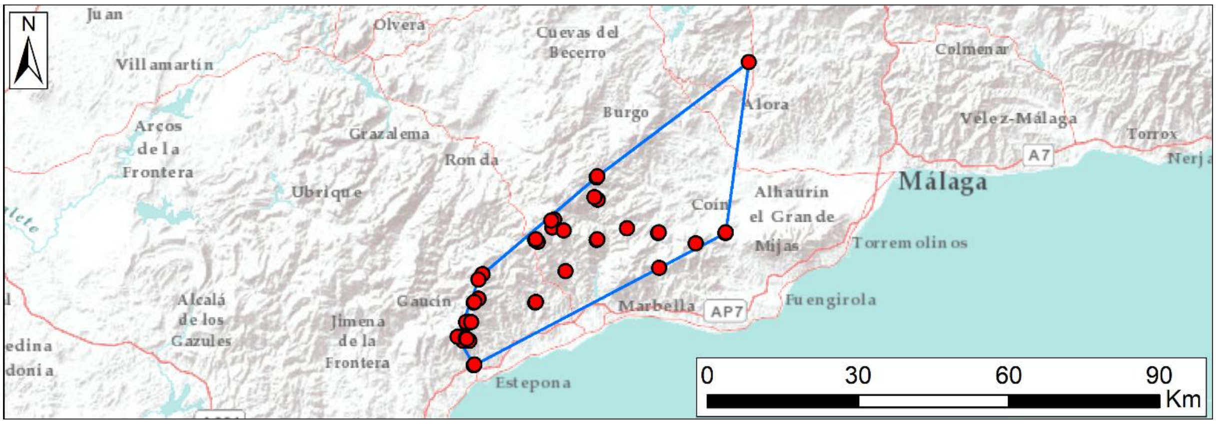

Group 7. *Silene fernandezii*

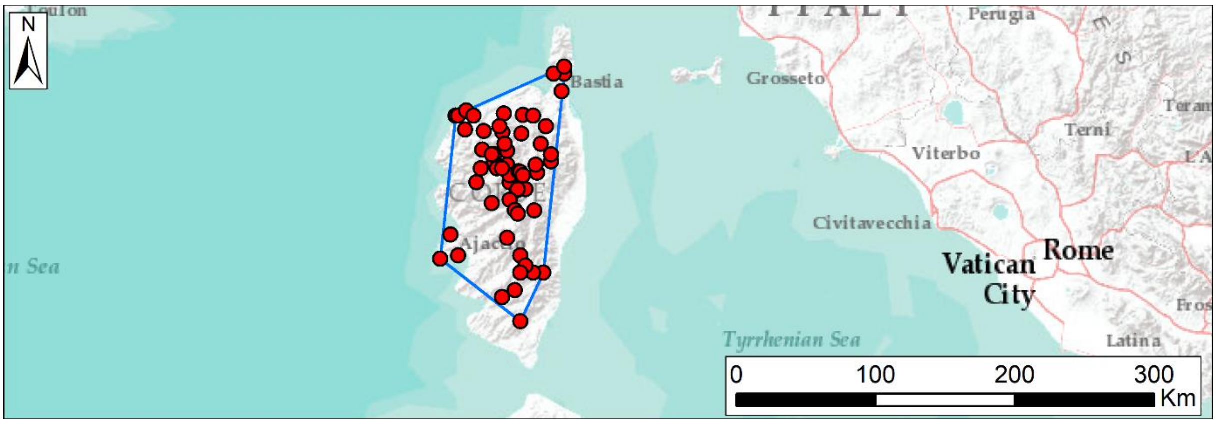

Group 8. *Silene nodulosa* (Corsica)

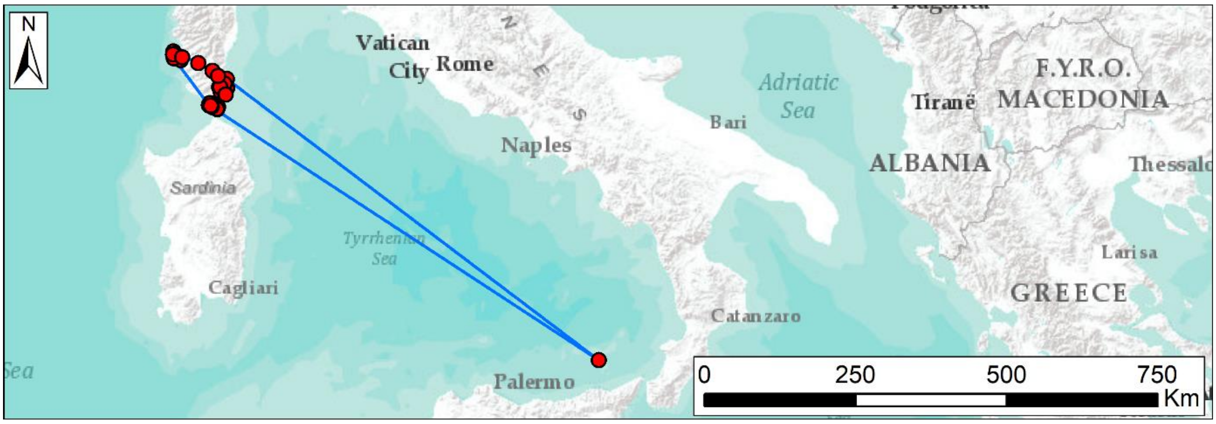

Group 9. *Silene velutina* and *S. hicesiae*

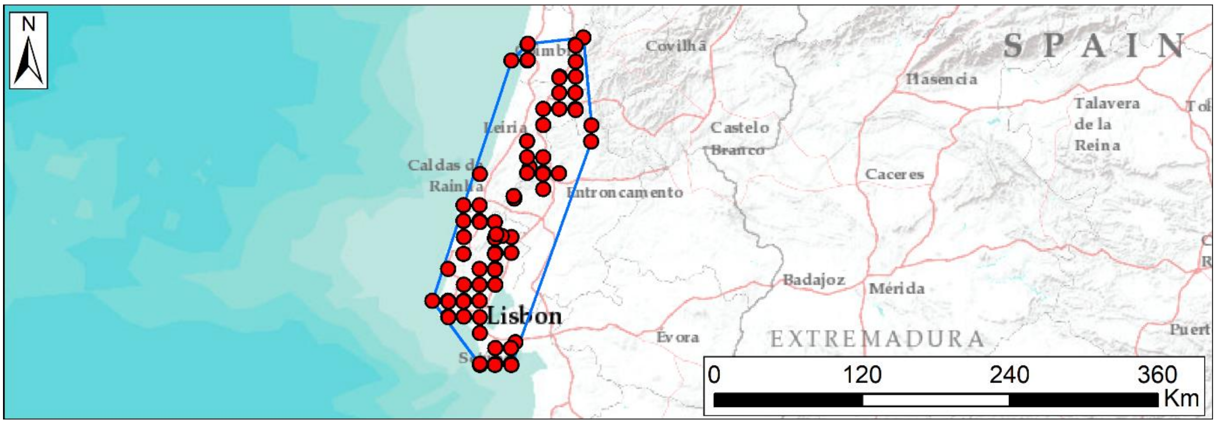

Group 10. *Silene coutinhoi*

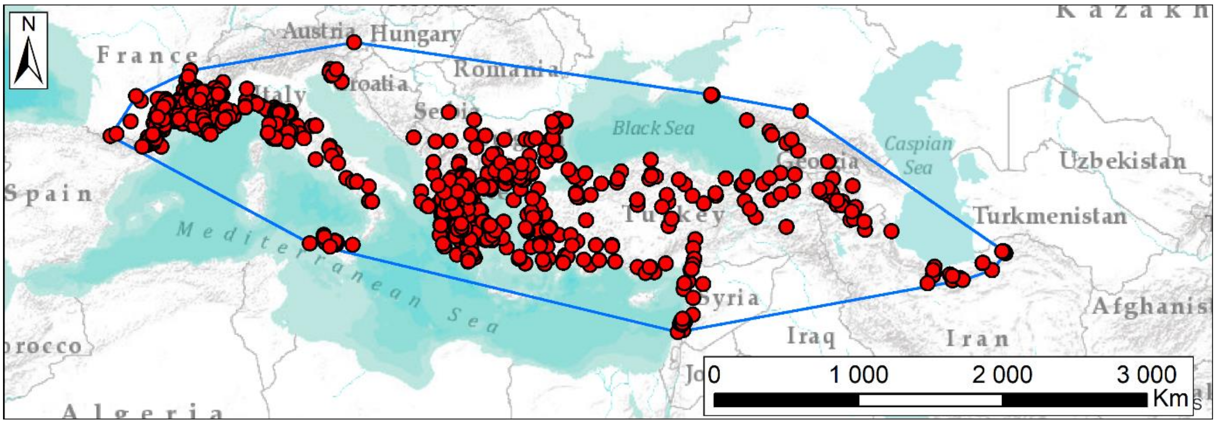

Group 11. *Silene italica*, *S. damboldtiana*, *S. badaroi* and *S. oenotriae*

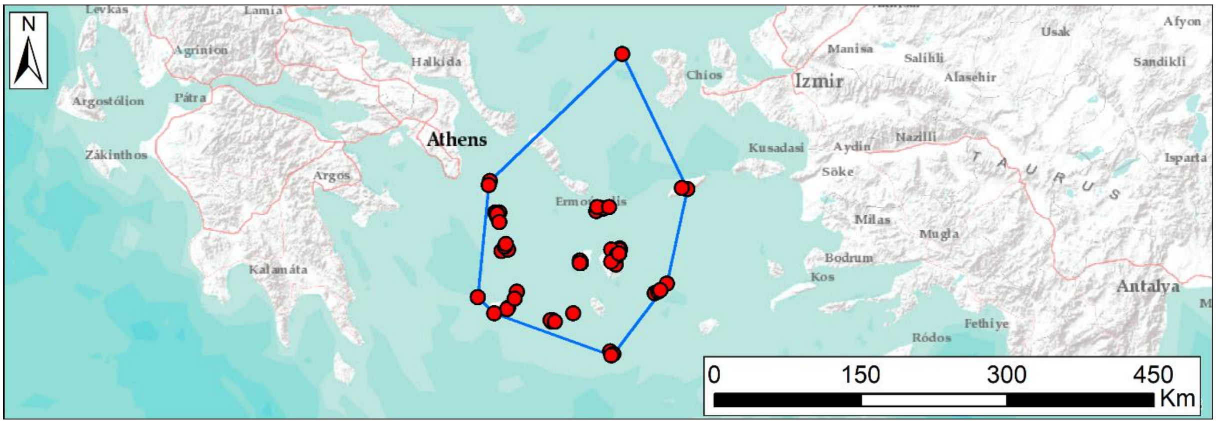

Group 12. *Silene cythnia*

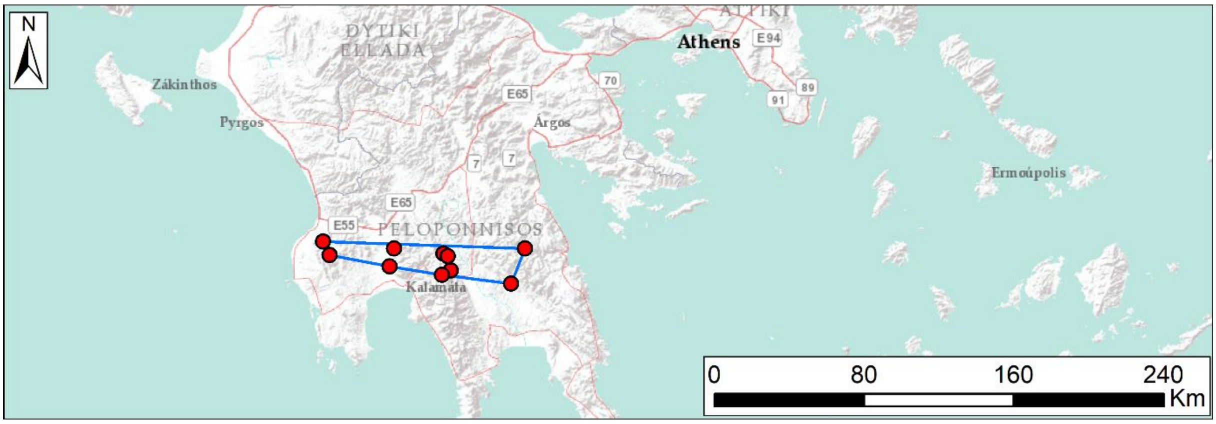

Group 13. *Silene goulimyi*

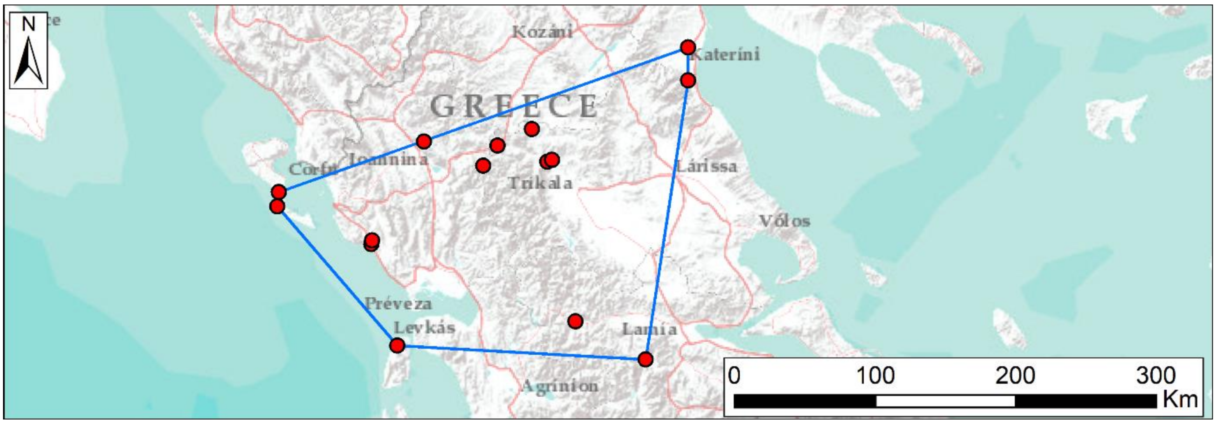

Group 14. *Silene niederi*

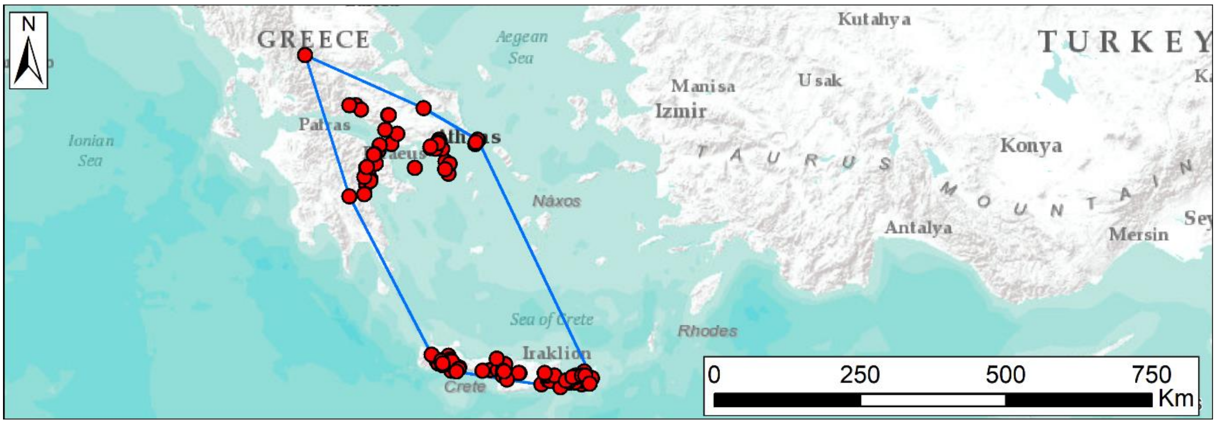

Group 15. *Silene sieberi* and *S. spinescens*

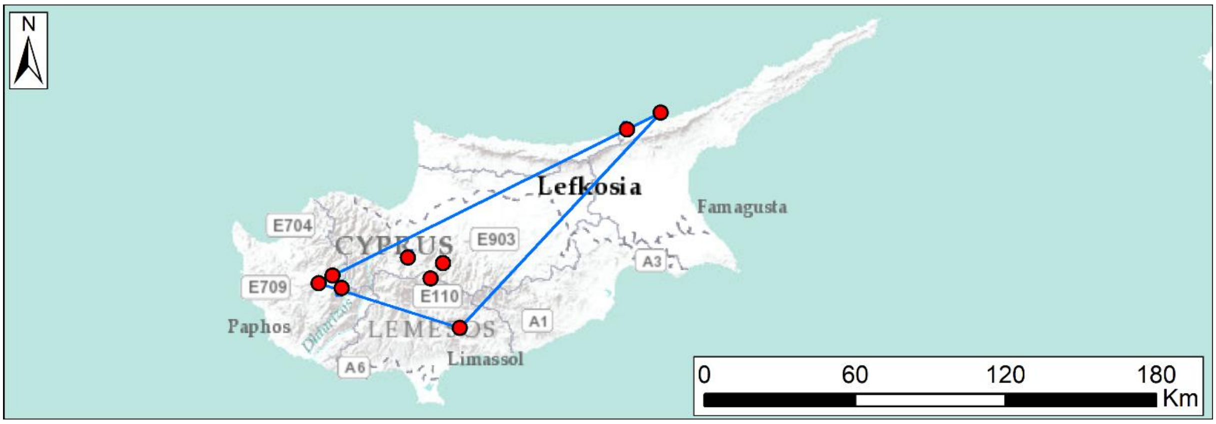

Group 16. *Silene galataea*
